# Photodynamic Modulation of Type 1 Interferon Pathway on Melanoma Cells Promotes Dendritic Cell Activation

**DOI:** 10.3389/fimmu.2019.02614

**Published:** 2019-11-08

**Authors:** María Julia Lamberti, Fátima María Mentucci, Emiliano Roselli, Paula Araya, Viviana Alicia Rivarola, Natalia Belén Rumie Vittar, Mariana Maccioni

**Affiliations:** ^1^Departamento de Biología Molecular, Facultad de Ciencias Exactas, Físico-Químicas y Naturales, Instituto de Biotecnología Ambiental y Salud, Consejo Nacional de Investigaciones Científicas y Técnicas, Universidad Nacional de Río Cuarto, Córdoba, Argentina; ^2^Departamento de Bioquímica Clínica, Facultad de Ciencias Químicas, Centro de Investigaciones en Bioquímica Clínica e Inmunología, Consejo Nacional de Investigaciones Científicas y Técnicas, Universidad Nacional de Córdoba, Córdoba, Argentina

**Keywords:** photodynamic therapy, IFN-1, dendritic cells, melanoma, immunotherapy

## Abstract

The immune response against cancer generated by type-I-interferons (IFN-1) has recently been described. Exogenous and endogenous IFN-α/β have an important role in immune surveillance and control of tumor development. In addition, IFN-1s have recently emerged as novel DAMPs for the consecutive events connecting innate and adaptive immunity, and they also have been postulated as an essential requirement for induction of immunogenic cell death (ICD). In this context, photodynamic therapy (PDT) has been previously linked to the ICD. PDT consists in the administration of a photosensitizer (PS) and its activation by irradiation of the affected area with visible light producing excitation of the PS. This leads to the local generation of harmful reactive oxygen species (ROS) with limited or no systemic defects. In the current work, Me-ALA inducing PpIX (endogenous PS) was administrated to B16-OVA melanoma cells. PpIX preferentially localized in the endoplasmic reticulum (ER). Subsequent PpIX activation with visible light significantly induced oxidative ER-stress mediated-apoptotic cell death. Under these conditions, the present study was the first to report the *in vitro* upregulation of IFN-1 expression in response to photodynamic treatment in melanoma. This *IFN-*α/β transcripts upregulation was concurrent with IRF-3 phosphorylation at levels that efficiently activated STAT1 and increased ligand receptor (cGAS) and ISG (CXCL10, MX1, ISG15) expression. The IFN-1 pathway has been identified as a critical molecular pathway for the antitumor host immune response, more specifically for the dendritic cells (DCs) functions. In this sense, PDT-treated melanoma cells induced IFN-1-dependent phenotypic maturation of monocyte-derived dendritic cells (DCs) by enhancing co-stimulatory signals (CD80, MHC-II) and tumor-directed chemotaxis. Collectively, our findings showed a new effect of PDT-treated cancer cells by modulating the IFN-1 pathway and its impact on the activation of DCs, emphasizing the potential relevance of PDT in adoptive immunotherapy protocols.

## Introduction

Cutaneous melanoma is the neoplasm originated from the melanocytes of the epidermis, and, although it corresponds to only 4% of skin related cancers, it is the causal agent for 80% of deaths from dermatological cancer (1). Unlike other tumor types, melanoma incidence and its mortality rate increased each year, an event associated with excesses in sun exposure and the progressive loss of the ozone layer (2). When surgical excision is performed on tumors with early diagnosis, the average survival rate at 10 years is 80%. However, in the case of metastatic melanoma, survival decreases to <10% (3). Therefore, the major challenge focuses on designing new therapies to treat melanoma in advanced stages with systemic dispersion. In this sense, immunotherapy emerges as a promising therapeutic option that involves therapeutic strategies with the common aim of enhancing the strengthens of the patient's immune system to advance upon tumors (4, 5). These systemic treatments for melanoma, approved or in experimental phase, include the administration of cytokines and other non-specific immunostimulatory molecules (IL-2, IFN-α2), active immunization (vaccination) with tumor cells, dendritic cells (DCs) or other molecules (recombinant antigens), adoptive transfer of T lymphocytes and monoclonal antibodies against immune checkpoint inhibitors (anti-CTLA-4, anti-PD1, anti-PDL1) (6).

Type I interferons are pleiotropic polypeptides classified according to the activity, structure and type of receptor to which they are bound in IFN-α, β, κ, ω, τ, and ε. Among them, IFN-α and IFN-β are the best characterized in terms of the stimulation of innate and adaptive immunity induced through autocrine and paracrine binding to the common IFNAR1/2 receptor. Previous reports indicated that type I IFNs (IFN-1) have an essential role in both basal and therapeutic-induced immune responses to cancer (7).

Clinical studies showed that high-dose IFN-α2 treatment was favorable for prolonging patient survival, therefore the exogenous administration of this was authorized as an adjuvant treatment for melanoma in 1996 (8). Unfortunately, high-dose treatment is also linked with adverse effects that can be reduced with lower doses, but they do not offer the same therapeutic good outcome (9). On the other hand, IFN-β treatment demonstrated limited efficacy and high toxicity for the treatment of metastatic melanoma (10, 11).

Studies about treatment of melanoma with recombinant type I IFN are ongoing and aim to develop more efficient methods of administration, design optimal treatment regimens, and identify the patient populations that are most likely to benefit. Nonetheless, given their antitumor immune-promoting activity, a variety of stimuli that induce the endogenous expression of IFN-1 are currently evaluated as promising adjuvants in vaccines. In fact, contrasting with the traditional adjuvants like aluminum compounds, which mainly promote humoral immune responses, IFN-α/β is a very effective tool to enhance cell-mediated immunity (12). Therefore, complementary efforts have focused on developing and identifying novel stimuli capable of promoting the IFN-1 pathway.

In this context, the molecular mechanisms subjacent the promotion of an immunogenic modality of cell death, that is, immunogenic cell death (ICD) have been elucidated. ICD includes spatiotemporally coordinated changes in the cell surface and the secretion of soluble mediators. Such signals are recognized by innate receptors expressed by dendritic cells to stimulate the antigenic presentation to T cells. These exposure/released danger signals, called damage-associated molecular patterns (DAMPs), include, but are not limited to, several innate immune stimulators, such as surface-exposed “eat me” signals (e.g., calreticulin, CRT), “find me” signals (e.g., ATP) and other factors (e.g., HMGB1) (13–15). Recently, IFN-1 signaling has been postulated as an essential requirement for ICD (16).

In the last decade, several investigations have analyzed the ability of conventional antitumor to promote ICD, in order to optimize their clinical use and to rationalize their application instead of more immunosuppressive drugs (17). In this context, photodynamic therapy (PDT) has been previously linked to the ICD. PDT is a well-known two-stage procedure. First, non-toxic photosensitizer drug (PS) is administrated and accumulates in tumor sites. After administration of the photosensitizer agent (PS), tumor loci are irradiated with a PS-exciting light of specific wavelength. None of these are independently toxic, but together produce a photochemical reaction, turning molecular oxygen into reactive oxygen species (ROS), which act directly on tumor cells or indirectly by damaging tumor-associated vasculature (18–22).

PDT has been associated with some of the main DAMPs involved in immunogenic cell death (23), such as CRT (24, 25), ATP (26), and HMGB1 (24). However, the relevance of PDT-mediated tumor cell death and its relationship with the IFN-1 pathway remain to be determined.

In the current study, we demonstrated that photodynamic treatment of melanoma cells *in vitro* resulted in IFN-α/β upregulation. Correspondingly, DCs co-cultured with PDT-treated tumor cells showed a potent IFN-1-dependent phenotypic and functional maturation. Taken together, these results delineate a novel photomodulated mechanism with potential application to prepare vaccines using *ex vivo* stimulated DC cultures with photosensitized tumor cells, which ultimately could lead to more effective immunotherapeutic interventions.

## Materials and Methods

### Reagents and Plasmids

LPS from Escherichia coli 055:B5, Methyl-aminolevulinic acid (Me-ALA), Doxorubicin, N-acetyl-L-cysteine (NAC), and BAPTA-AM were from Sigma Aldrich. The plasmid pEYFP-Mito (mitochondrial marker) (27) was from Clontech. The plasmid pEYFP-C1-KDEL-GFP (28) (endoplasmic reticulum marker) was kindly provided by Dr. Sergio Grinstein (University of Toronto, Canada). The plasmid pCRT-EGFP (29) (Green fluorescent protein-tagged calreticulin) was kindly provided by Dra. Marta Hallak (CIQUIBIC, Argentina).

### Cell Culture

B16-OVA murine melanoma cells were grown, as previously described, “in complete medium DMEM (Dulbecco's modified Eagle medium high glucose 1X, Gibco) supplemented with 10% v/v fetal bovine serum (FBS) (PAA Laboratories), 1% v/v glutamine (GlutaMAXTM 100X Gibco), 1% v/v antibiotic (Penicillin 10,000 units/mL–streptomycin 10,000 μg/mL Gibco) and 1% v/v of sodium pyruvate 100 mM (Gibco). Cells were maintained in 5% CO_2_ and 95% air at 37°C in a humidified incubator. Stock cultures were stored in liquid nitrogen and used for experimentation within 5–7 passages” (30).

### Animals

C57BL/6 were purchased from Universidad Nacional de La Plata (Buenos Aires, Argentina) and IFNAR1^−^/^−^ were kindly provided by CIBICI-UNC (Cordoba, Argentina, purchased from Jackson Laboratory) (31). Animals were maintained under specific pathogen-free conditions at the Animal Resource Facility of Facultad de Ciencias Exactas, Físico-Químicas y Naturales (Universidad Nacional de Río Cuarto) in accordance with the experimental ethics committee guidelines. Experiments were in compliance with the Guide for the Care and Use of Laboratory Animals published by the NIH and approved by the Comité de Ética de la Investigación (COEDI) from Universidad Nacional de Río Cuarto.

### Photodynamic Treatment

As previously described, B16-OVA cells monolayers “were washed twice with PBS to remove all traces of FBS and then incubated with 5-methylaminolevulinic acid (Me-ALA, Sigma) in medium without FBS for 4 h to allow the endogenous generation of the photosensitizer PpIX. After Me-ALA incubation, tumor cells were irradiated at room temperature with monochromatic light source (636 ± 17 nm) using a MultiLED system (coherent light). The fluence rate was 0.89 mW/cm^2^, as measured by Radiometer Laser Mate-Q. Drug solution was then removed and replaced with fresh medium” (30).

### Photosensitizer Localization Assay

B16-OVA cells were seeded on glass coverslips in a 24-well plate and allowed to attach overnight. Next, cells were transfected with pEYFP-Mito (mitochondrial marker) (27) or pEYFP-C1-KDEL-GFP (endoplasmic reticulum marker) (28). Transient transfections were performed using FuGENE® HD Transfection Reagent (Roche) according to the manufacturer's instructions (32). The following day, cells were washed twice with PBS to remove all traces of FBS and then incubated with 5-methylaminolevulinic acid (1 mM) in medium without FBS for 4 h to allow the endogenous generation of the photosensitizer PpIX. Next, they were fixed with paraformaldehyde (PAF) 4% for 20 min, and the cell nuclei were stained with Hoechst (HÖ) for visualization. The fluorescence of PpIX (red), organelles (green) and nuclei (blue) was observed by confocal microscopy (Olympus FV1000 Spectral confocal microscope, CIQUIBIC-UNC-CONICET). The co-localization is evidenced in yellow color. The analysis of the images was carried out using the free ImageJ 1.42q software (plugging Coloc 2), and the correlation was quantified through the Pearson coefficient (r).

### Calreticulin (CRT) Localization Assay

B16-OVA cells were seeded in a 24-well plate and allowed to attach overnight. Next, cells were transfected with pCRT-EGFP (29) (Green fluorescent protein-tagged calreticulin). Transient transfections were performed using FuGENE® HD Transfection Reagent (Roche) according to the manufacturer's instructions (32). The following day, cells were washed twice with PBS to remove all traces of FBS and then incubated with 5-methylaminolevulinic acid (0.3 mM) in medium without FBS for 4 h to allow the endogenous generation of the photosensitizer PpIX. After Me-ALA incubation, tumor cells were irradiated with a light dose of 0.5 J/cm^2^. The localization of CRT was observed 1 h after treatment on an inverted Carl Zeiss fluorescence microscope (UNRC) coupled to a high resolution monochromatic digital camera. The analysis of the images was carried out using the free ImageJ 1.42q software.

### Cell Viability Assay

As previously described, “cell viability was evaluated by 1-(4,5-dimethylthiazol-2-yl)-3,5-diphenylformazan (MTT) assay, which is reduced by mitochondrial dehydrogenases of viable cells to non-water-soluble violet formazan crystals. Twenty-four hours post-PDT, MTT solution (5 mg/ml in phosphate buffer saline, PBS) was added for 3 h (dilution rate: 1/10). Then, dimethyl sulfoxide (DMSO) was added to lyse the cells and solubilize the precipitated formazan product. Optical density of the resulting solution of formazan salt was read at 540 nm using ELISA reader plate (Thermo Scientific, Multiskan FC) (33).

### Analysis of Apoptosis Rate by Annexin V-FITC/PI Assay

Twenty-four hours after treatment, the percentage of apoptotic cells was assessed using a standard flow cytometry Annexin-V-FITC binding assay (BD Pharmingen). Briefly, cells were disaggregated by trypsin digestion and washed with PBS. The pellet was incubated at room temperature with 5 μg/ml Annexin V-FITC, 5 μg/ml propidium iodide (PI) and binding buffer for 15 min in the dark. Annexin V and PI fluorescence were measured using a Millipore Guava Easycyte 6 2L cytometer. According to the manufacturer's instructions, “cells that stain positive for FITC Annexin V and negative for PI are undergoing apoptosis. Cells that stain positive for both FITC Annexin V and PI are either in the end stage of apoptosis, are undergoing necrosis, or are already dead. Cells that stain negative for both FITC Annexin V and PI are alive and not undergoing measurable apoptosis.” Data was analyzed using FlowJo 10.0.7 software.

### Quantitative Real Time RT-PCR

Total RNA was extracted using Trizol Reagent (Life Technologies) and M-MLV reverse transcriptase was used to generate cDNA (Promega). Target transcripts were quantified by real time qRT-PCR (Stratagene Mx3000PRO) using the Mx3000P software (34). Experiments were performed using SYBR Green PCR Master Mix (Applied Biosystems) (35). The gene-specific primers were designed with the Primer BLAST software: GAPDH: Forward: TGCACCACCAACTGCTTAG–Reverse: GGATGCAGGGATGATGTTC; IFN-α: Forward: TCTGATGCAGCAGGTGGG–Reverse: AGGGCTCTCCAGACTTCTGCTCTG; IFN-β: Forward: GCACTGGGTGGAATGAGACT–Reverse: AGTGGAGAGCAGTTGAGGACA; RIG1: Forward: AAGAGCCAGAGTGTCAGAATCT–Reverse: AGCTCCAGTTGGTAATTTCTTGG; TLR3: Forward: GTGAGATACAACGTAGCTGAACT–Reverse: TCCTGCATCCAAGATAGCAAGT; MDA5: Forward: AGATCAACACCTGTGGTAACACC–Reverse: CTCTAGGGCCTCCACGAACA; cGAS: Forward: GAGGCGCGGAAAGTCGTAA–Reverse: TTGTCCGGTTCCTTCCTGGA; ISG15: Forward: GGTGTCCGTGACTAACTCCAT–Reverse: TGGAAAGGGTAAGACCGTCCT; CXCL10: Forward: AGTGCTGCCGTCATTTTCTG–Reverse: ATTCTCACTGGCCCGTCAT; MX1: Forward: AGACTTGCTCTTTCTGAAAAGCC–Reverse: GACCATAGGGGTCTTGACCAA. Specificity was verified by melting curve analysis. Fold change in gene expression was calculated according to the 2^−ΔΔ*Ct*^ method. Each sample was analyzed in triplicate. No amplification was observed in PCR reactions containing water.

### Western Blot

As previously described, “total cell lysates were extracted with lysis buffer containing 20 mM HEPES pH 7.5; 1.5 mM KCl; 1 mM EDTA; 1 mM EGTA; 0.15% Triton-X100; 1 mM PMSF; 1 mM DTT; and a cocktail of protease inhibitors (Sigma). The protein content of the lysate was measured using BCA protein assay reagent (Pierce). Aliquots containing an equal amount of protein (30 μg) were separated by SDS-PAGE and then transferred onto PVDF membranes (Sigma). Blots were blocked with 5% non-fat dry milk in PBS Tween 0.1% (PBST) and then incubated with primary antibodies overnight” (30): anti-phosphoIRF3 antibody (Cell Signaling−4947), anti-phospo-STAT1 (Cell Signaling−9167), anti-STAT1 (Cell Signaling−9172), anti-α-Tubulin (Cell Signaling−2144). Next, blots were incubated with corresponding horseradish peroxidase–conjugated IgG secondary antibody (anti-rabbit or anti-mouse, Cell Signaling). Detection of immunoreactive bands detection was carried out using the enhanced chemiluminescence (ECL) kit (Amersham) according to the manufacturer's instructions.

### Dendritic Cell Differentiation From Bone Marrow Precursors

Dendritic cells (DCs) were obtained from bone marrow of C57BL/6 and IFNAR1^−^/^−^ mice as described previously (36). Briefly, isolated bone marrow cells from femurs and tibiae were cultured for 7 days at a density of 3 ×10^6^ per 10-cm dish (10 ml) in RPMI medium supplemented with 10% FBS (PAA Laboratories), 1% v/v glutamine (GlutaMAXTM 100X Gibco), 1% v/v antibiotic (Penicillin 10,000 units/mL–streptomycin 10,000 μg/mL Gibco), 1% v/v of sodium pyruvate 100 mM (Gibco) and GM-CSF (10% J558-conditioned medium v/v), hereafter termed “complete differentiation medium,” in 5% CO_2_ and 95% air at 37°C in a humidified incubator. On day 3, floating cells were discarded and fresh complete differentiation medium was added. Cells were further differentiated for an additional 4 days. Floating and attached cells were separately examined for their surface marker expressions, and we obtained attached cells in this study by scraping after gently washing the culture plates with warm PBS twice. More than 80% of harvested cells were immature dendritic cells (imDCs) CD11c^+^.

### Transwell Migration Assays

WT or IFNAR^−/−^ CD11c^+^ imDCs (2 ×10^5^ cells) were loaded in their own “complete differentiation medium” in the upper chamber of a Transwell apparatus (5-μm pore size; Cornings, Lowell, MA), while B16-OVA (TCs) or PDT-treated B16-OVA (PDT-TCs) (3 ×10^4^ cells) were seeded in the lower chamber. After 16 h at 37°C, DCs that have migrated through the membrane toward the tumor stimuli and attached on the underside of the membrane were stained with Hoechst dye for 1 h. After that, epifluorescence images were taken using an inverted Carl Zeiss fluorescence microscope (UNRC) and migrating cells were counted in different fields of view (37).

### Dendritic Cell Maturation

For dendritic cells maturation analysis, WT or IFNAR^−^/^−^ imDCs were co-cultured with B16-OVA (TCs) or with PDT-treated B16-OVA (PDT-TCs) for 24 h at a 1:1 ratio. As positive control, imDCs were exposed to LPS (0.5 μg/mL) for 24 h. CD86 and MHC-II were used as DC maturation markers evaluated on the CD11c^+^ population (36).

### Flow Cytometry

Surface staining of single-cell suspensions of dendritic cells was performed using standard protocols (31) and analyzed on a Millipore Guava Easycyte 6 2L cytometer. Data analysis was conducted using FlowJo 10.0.7 software. The following were obtained from BioLegend: anti-CD11c-APC (147309), anti-MHC-II-PerCP-Cy5.5 (107626), and anti-CD86-PeCy5 (105014).

### Statistics

Data handling, analysis and graphic representation (all shown as mean ± SEM) were performed using Prism 7.0 (GraphPad Software). Statistical data are informed in the corresponding figure legend.

## Results

### ER-Associated Cell Death Promoted by Photodynamic Therapy

**Figure 1:**
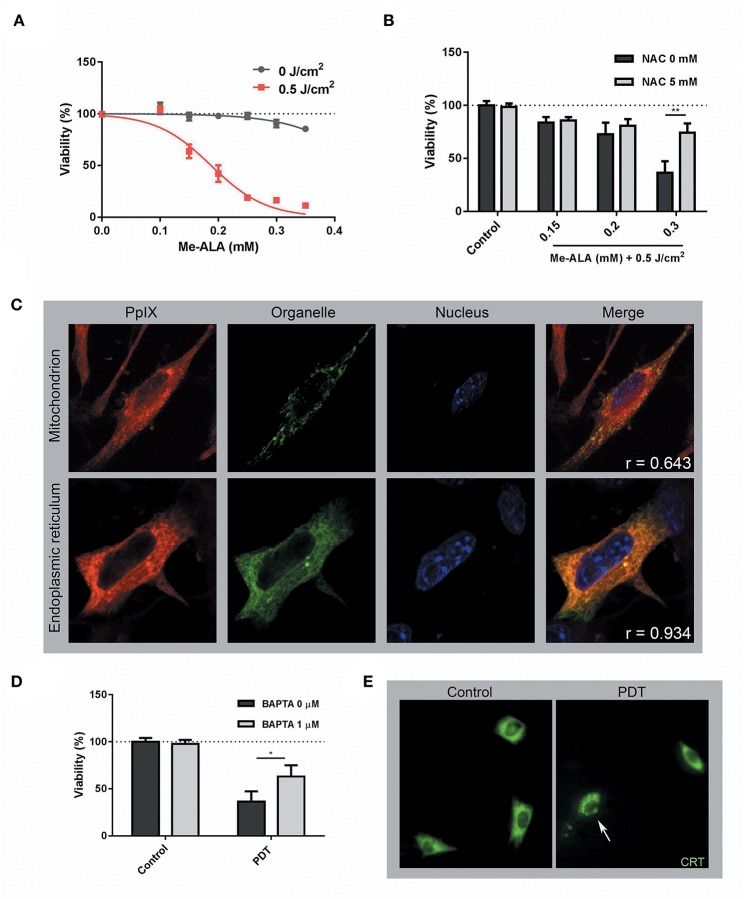
Photodynamic Therapy induced ER-associated cell death and CRT mobilization on melanoma cells. **(A)** B16-OVA cells were incubated with increasing concentrations of the pro-drug Me-ALA (0–0.35 mM) for 4 h and then were irradiated with visible light (λ: 635 ± 17 nm, light dose: 0.5 J/cm^2^). At 24 h post-treatment, cell viability was evaluated through the MTT assay and expressed as a percentage with respect to the non-treated control (dotted line: 100% viability). **(B)** B16-OVA cells were incubated with Me-ALA (0.1, 0.2, and 0.3 mM) in the presence or absence of NAC (5 mM) for 4 h and then exposed to irradiation (0.5 J/cm^2^). Viability was evaluated by MTT assay 24 h post-PDT and referred to non-treated conditions (dotted line: 100% viability). Data are mean ± SEM of three independent experiments. ^**^p < 0.01 vs. control group (NAC 0 mM, gray bars), Two-Way ANOVA Bonferroni post-test. **(C)** B16-OVA cells transfected with pEYFP-Mito (mitochondrial marker) and pEYFP-C1-KDEL-GFP (endoplasmic reticulum marker) were incubated for 4 h with the Me-ALA drug (1 mM). Next, they were fixed with paraformaldehyde (PAF) 4% for 20 min and the cell nuclei were stained with Hoechst (HÖ) for visualization. The fluorescence of PpIX (red), organelles (green), and nuclei (blue) was observed by confocal microscopy. The co-localization is evidenced in yellow color. The analysis of the images was carried out using the free ImageJ 1.42q software (plugging Coloc 2) and the correlation was quantified through the Pearson coefficient (r). **(D)** B16-OVA cells were subjected to high dose PDT (Me-ALA 0.3 mM + 0.5 J/cm^2^) in the presence or absence of BAPTA-AM (1 μM) for 4 h. Viability was evaluated by MTT assay 24 h post-PDT and referred to non-treated conditions (dotted line: 100% viability). Data are mean ± SEM of three independent experiments. ^*^p < 0.05 vs. control group (BAPTA 0 μM, gray bars), Two-Way ANOVA Bonferroni post-test. **(E)** B16-OVA cells transfected with pCRT-EGFP [green fluorescent protein-tagged calreticulin (CRT)] were subjected to high dose PDT (Me-ALA 0.3 mM + 0.5 J/cm^2^). The fluorescence of CRT (green) was observed by epifluorescence microscopy 0.5 h post-treatment. CRT translocation is marked with a narrow. The analysis of the images was carried out using the free ImageJ 1.42q software.

By definition, “ICD inducers must be cytotoxic and provoke cell death above a minimal threshold level” (15). Therefore, we initially examined the ability of PDT to elicit melanoma cell death. In this context, B16-OVA were incubated during 4 h with the prodrug Me-ALA to allow the generation of the photosensitizer PpIX. Upon red-light activation (0.5 J/cm^2^), variable cell toxicity dependent on the pro-drug concentration (0.1–0.35 mM) was observed (Figure 1A). No damage was induced by the prodrug Me-ALA *per se* or by red-light irradiation alone (Figure 1A). As expected, the antioxidant NAC (30) reversed the cytotoxic effect of high-dose PDT (Figure 1B). To determine the organelles in which PpIX was located, we performed co-localization experiments with mitochondria and endoplasmic reticulum markers. We observed that PpIX displayed preferential endoplasmic reticulum (ER) localization (Figure 1C). Pre-incubation of melanoma cells with the calcium chelator BAPTA-AM (38) inhibited PDT-induced cell death (Figure 1D), indicative of ER stress associated with photodynamic effect. The ER response to stress is accompanied by translocation of danger signals to the cell surface (38). CRT is the most abundant protein in the ER lumen which translocates to cell surface in response to stress-mediated dying cells (39). Here, 72.3% of photosensitized melanoma cells exhibited the typical “patches” (40) of anterograde intracellular transport of CRT, suggesting that PDT also modulates CRT mobilization (Figure 1E).

### Enhancement of IFN-1 Expression Mediated by Photodynamic Treatment

**Figure 2:**
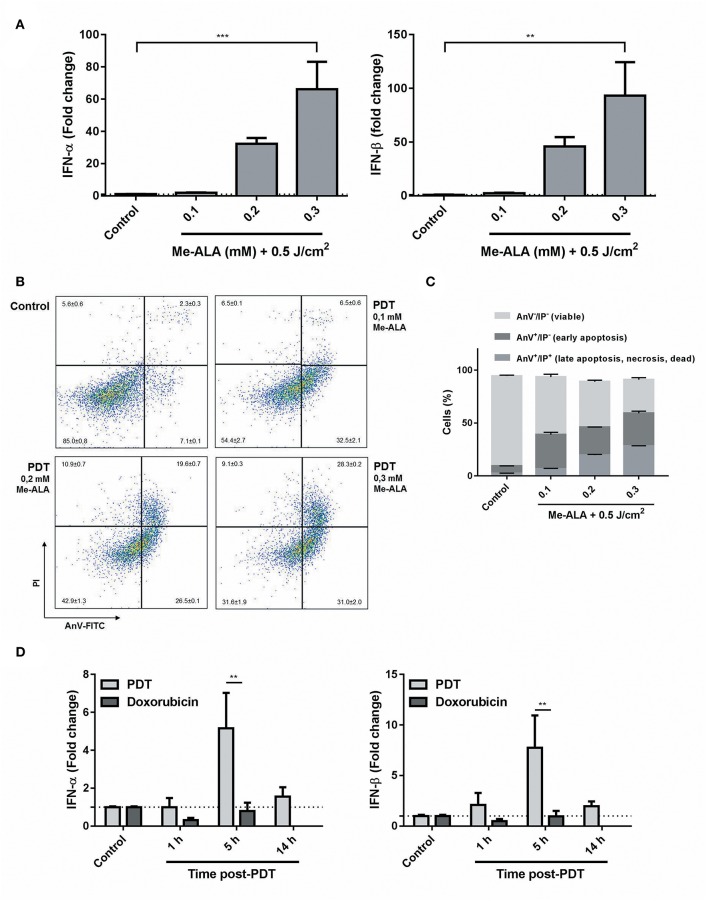
Photodynamic therapy as a novel inductor of IFN-1 expression on melanoma cells. **(A)** B16-OVA cells were incubated with Me-ALA (0.1, 0.2, and 0.3 mM) for 4 h and then were irradiated with visible light (0.5 J/cm^2^). Quantification of mRNA expression of IFN-1α (right) and IFN-β (left) was performed 5 h after treatment by RTqPCR and normalized with respect to the non-treated control (dotted line: 1). Data are mean ± SEM of three independent experiments. ^**^p < 0.01, ^***^p < 0.05 vs. control group (untreated cells), One-Way ANOVA Bonferroni post-test. **(B)** Type of cell death was evaluated using Annexin V/PI staining by flow cytometry. The data generated by flow cytometry were plotted in two-dimensional dot plots in which PI is represented vs. Annexin V-FICT. **(C)** Viable cells (Annexin V^−^/PI^−^), undergoing (early) apoptotic cells (Annexin V^+^/PI^−^) and dead, necrotic or late (end-stage) apoptotic cells (Annexin V^+^/PI^+^) were quantified using FlowJo 10.0.7 software. **(D)** B16-OVA cells were subjected to high dose PDT (Me-ALA 0.3 mM + 0.5 J/cm^2^) or doxorubicin (30 μM). Quantification of mRNA expression of IFN-1-α (right) and IFN-β (left) was performed 1, 5, and 14 h after treatment by RTqPCR and normalized with respect to the non-treated control (dotted line: 1). Data are mean ± SEM of three independent experiments. ^**^p < 0.01 vs. PDT group (gray bars), Two-Way ANOVA Bonferroni post-test.

Until now, PDT had been associated with CRT (24, 25), ATP (26), and HMGB1 (24) exposition and/or release, but there was no evidence for type I interferon pathway regulation. Having shown that CRT was translocated through photodynamic stimuli (Figure 1E) and given the connection between this chaperone and the modulation of IFN-1 (41), levels of *ifn-*α/β mRNA were measured in dying cells undergoing anticancer PDT. B16-OVA were exposed to Me-ALA (0.1, 0.2, and 0.3 mM) and then irradiated with red light (0.5 J/cm^2^). Interestingly, a significant increase in IFN-1 transcription was detected in melanoma cells as early as 5 h following high-dose photosensitization (Figure 2A). In addition, as the doses of Me-ALA increased, the frequency of apoptotic cells (both early and late apoptotic cells) (Figures 2B,C) and the expression of IFN-α/β also augmented (Figure 2A), suggesting that an autocrine effect of IFN-1 could be playing a role in inducing apoptosis. Next, we exposed melanoma cells to a lethal dose of PDT (Me-ALA 0.3 mM + 0.5 J/cm^2^) or doxorubicin (30 μM), a relevant chemotherapeutic agent bona fide ICD inducer (16, 42), analyzing IFN-1 regulation in a time-course experiment. Notably, PDT was a strong IFNα/β inducer 5 h post-PDT; in contrast the significant upregulation of IFN-α/β was absent in those subjected to doxorubicin (Figure 2D). Overall, we provide here experimental data regarding specific *in vitro* apoptotic lethal conditions of PDT that strongly induced IFN-1 in B16-OVA cells.

### Photodynamic Autocrine Modulation of Type 1 Interferon Pathway

**Figure 3:**
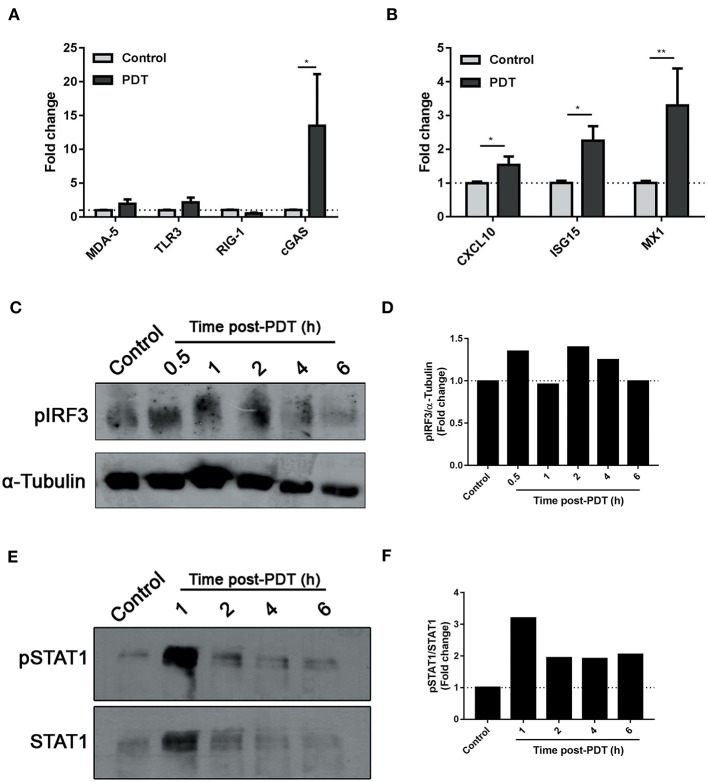
Modulation of IFN-1 pathway by photodynamic therapy. B16-OVA cells were incubated with Me-ALA (0.3 mM) for 4 h and then were irradiated with visible light (λ: 635 ± 17 nm, light dose: 0.5 J/cm^2^) (PDT). Non-treated cells were used as “Control.” **(A)** Quantification of mRNA expression of receptors MDA-5, TLR3, RIG-1, and cGAS was performed 14 h after treatment by RTqPCR and normalized with respect to the non-treated control (dotted line: 1). Data are mean ± SEM of three independent experiments. ^*^p < 0.05 vs. control group (untreated cells, gray bars), Two-Way ANOVA Bonferroni post-test. **(B)** Quantification of mRNA expression of ISGs CXCL10, ISG15, and MX1 was performed 14 h after treatment by RTqPCR and normalized with respect to the non-treated control (dotted line: 1). Data are mean ± SEM of three independent experiments. ^*^p < 0.05, ^**^p < 0.01 vs. control group (untreated cells, gray bars), Two-Way ANOVA Bonferroni post-test. **(C)** Western blot was performed to detect phospho-IRF3 at 0.5–6 h post-treatment. The same membrane was stripped and reblotted for α-Tubulin as loading control. **(D)** Densitometric analysis performed with the ImageJ software represented the signal intensity of phospho-IRF3; the signal was normalized α-Tubulin intensity. **(E)** Western blot was performed to detect phospho-STAT1 at 1–6 h post-treatment. The same membrane was stripped and reblotted for total STAT1 as loading control. **(F)** Densitometric analysis performed with the ImageJ software represented the signal intensity of phospho-STAT1; the signal was normalized total STAT1 intensity.

Type I IFNs are cytokines of major importance for the innate antiviral response that have been recently associated to ICD (16). They are produced after recognition of nucleic acids by toll-like receptors (TLR3-7-8) or by cytoplasmic proteins, such as RIG-I like receptors (RIG-1, MDA-5) or the cyclic GMP-AMP synthase (cGAS), which activate adaptor proteins that culminate in IRF3 phosphorylation. IRF3 is a transcription factor that leads the expression of Type 1 interferons. After their secretion, IFN-α/β bind to their cognate receptor IFNAR1/2, triggering the phosphorylation of STAT transcription factors, and the consequent induction of hundreds of interferon-stimulated genes (ISGs) in the responding cells (7). Based on our findings (Figure 2), we decided to explore the mechanisms underlying the photodynamic modulation of IFN-1. After 14 h of PDT-stimulation, an upregulation of cGAS receptor, but not MDA-5, TLR3, or RIG-1, was detected (Figure 3A). Interestingly, a significant increase in the transcription of ISGs CXCL10, ISG15, and MX1 was observed (Figure 3B). As expected, induction of interferon regulatory factor (IRF) related genes was paralleled by phosphorylation of IRF3 0.5 h after photodynamic treatment (Figures 3C,D). The type I IFN autocrine loop was also manifested in our experimental setting, since STAT1 phosphorylation was evidenced 1 h after the initial PpIX photoactivation on tumor cells (Figures 2E,F). Collectively, these data suggest that Me-ALA-based PDT stimulates the production of type I IFN and this can act autocrinally augmenting the transcription of several interferon stimulated genes.

### IFN-1-Dependent Activation of Dendritic Cells Induced by PDT-Treated Melanoma Cells

**Figure 4:**
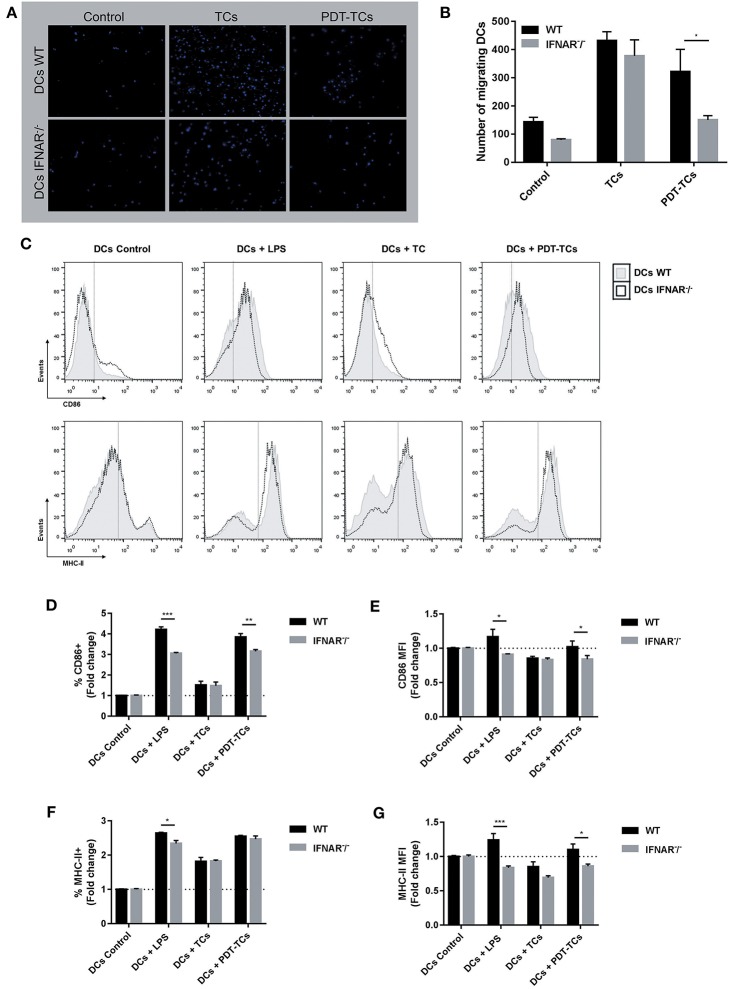
Phenotypic and functional maturation of dendritic cells mediated by IFN-1 upregulation on PDT-subjected melanoma cells. WT or IFNAR^−^/^−^ DCs were in the upper chamber of a Transwell apparatus while B16-OVA (TCs) or with PDT-treated B16-OVA (PDT-TCs) were seeded in the lower chamber. Complete growth media was used as “control.” **(A)** After 16 h at 37°C, DCs that have migrated through the membrane toward the tumor stimuli and attached on the underside of the membrane were stained with Hoechst dye for 1 h, and epifluorescence images were taken. **(B)** Migrating cells were counted in different fields of view. Data are mean ± SEM of three independent experiments. ^*^p < 0.05 vs. control group (WT dendritic cells, black bars), Two-Way ANOVA Bonferroni post-test. **(C)** WT (solid line, gray filled) or IFNAR^−^/^−^ DCs (dotted line, non-filled) were co-cultured with B16-OVA (TCs) or with PDT-treated B16-OVA (PDT-TCs) for 24 h in a 1:1 ratio. As positive control, DCs were exposed to LPS (0.5 μg/mL) for 24 h. CD86 and MHC-II were used as DCs maturation markers. Representative flow cytometry histograms were performed with FlowJo 10.0.7 software. **(D)** CD86^+^ cells were referred to untreated imDCs used as negative control (DCs Control, dotted line: 1). Data are mean ± SEM of three independent experiments. ^**^p < 0.01, ^***^p < 0.001 vs. control group (WT dendritic cells, black bars), Two-Way ANOVA Bonferroni post-test. **(E)** CD86 expression intensity of CD86^+^ cells was indicated by geometric mean (MFI, mean fluorescence intensity) referred to untreated imDCs used as negative control (DCs Control, dotted line: 1) Data are mean ± SEM of three independent experiments. ^*^p < 0.05 vs. control group (WT dendritic cells, black bars), Two-Way ANOVA Bonferroni post-test. **(F)** MHC-II^+^ cells were referred to untreated imDCs used as negative control (DCs Control, dotted line: 1) Data are mean ± SEM of three independent experiments. ^*^p < 0.05 vs. control group (WT dendritic cells, black bars), Two-Way ANOVA Bonferroni post-test. **(G)** MHC-II expression intensity of MHC-II^+^ cells was indicated by geometric mean (MFI) referred to untreated imDCs used as negative control (DCs Control, dotted line: 1). Data are mean ± SEM of three independent experiments. ^*^p < 0.05, ^***^p < 0.001 vs. control group (WT dendritic cells, black bars), Two-Way ANOVA Bonferroni post-test.

The spatiotemporally coordinated emission of specific DAMPs promotes the recruitment of DCs to sites of ongoing ICD and their capacity to prime an adaptive immune response (13). For this reason, we next examined whether IFN-1 detected in PDT-melanoma tumor cells (PDT-TCs) could act in a paracrine fashion on DC migration. Immature WT and IFNAR^−^/^−^ DCs were loaded into the upper chamber of transwells with growth media (Control), B16-OVA (TCs) or photosensitized B16-OVA (PDT-TCs) in the lower chamber. Although the absence of IFNAR did not affect the basal migration of dendritic cells or in response to untreated tumor cells, WT DCs migrated toward PDT-TCs in much greater numbers than IFNAR^−^/^−^ DCs (Figures 4A,B). The expression of cell-surface co-stimulatory molecules that are involved in DC maturation, such as CD86 and MHC-II, was assessed by flow cytometry after 24 h of DCs-TCs co-culture. Untreated imDCs were used as negative control (DCs control) and imDCs stimulated by lipopolysaccharide (DCs + LPS) were used as positive control. Interestingly, PDT-TCs were capable *per se* of significantly enhancing the maturation of WT DCs, which was partially abrogated when IFN-1 receptor was absent in DCs. Similar results were observed with the positive control of LPS treatment (Figures 4C–G). Taken together, these results indicated that apoptotic PDT on melanoma cells induces the production of type I IFNs, which in turn can promote an improvement in DC function.

## Discussion

The success of cancer treatments fundamentally relies on the synergic interaction between dying/dead cancer cells and immune cells. The ideal cancer therapeutic strategy should involve both direct cytotoxic action on tumor cells and immunostimulatory effects based on the immune recognition of molecular antigenic determinants on dying cells. However, to cause an immune response against malignant cells, the presence of tumor antigens is not enough. Also, such cells must emit danger signals, such as danger-associated molecular patterns (DAMPs) that work as adjuvants (43). In this context, several successful antitumor agents have demonstrated to be highly efficient in stimulating the emission of DAMPs by cancer cells, thus inducing ICD (15). Two categories have been proposed in order to classify ICD inducers based on their direct or indirect ability to cause endoplasmic reticulum (ER) stress leading to apoptotic cell death. The majority of ICD inducers, such as chemotherapeutic agents (oxaliplatin mitoxantrone, doxorubicin, and cyclophosphamide), shikonin, vorinostat, cardiac glycosides, among others, are categorized as type I ICD inducers that primarily target cytosolic proteins, plasma membranes, or nucleic proteins. They also induce ER stress via collateral effects. On the other hand, type II ICD inducers, such as hypericin-based PDT and coxsackievirus B3, preferentially target the ER. The quality and/or quantity of ER stress induced by ICD, also associated with oxidative stress, may define the ICD inducer properties, and it was demonstrated that type II ones are more efficient in terms of immunological antitumor ability (44).

In the last decades, several investigations have been devoted on the search of particular stress agents capable of provoking ICD in cancer cells. Photodynamic therapy (PDT), a regulatory approved cancer treatment, has the ability of inducing immunogenic apoptosis (45, 46). Here, we demonstrated that oxidative stress induced by PDT promoted apoptotic cell death (Figures 1, 2B,C). Following PDT, ROS exhibit a short half-life, thus they exert their effect close to their site of generation. Consequently, the precise subcellular localization of the PS within the cell influences the degree and the type of photodamage. The knowledge of PS localization is therefore important for choosing the most effective PS for each purpose (47). For this reason, we decided to evaluate the precise location of PpIX. Under our experimental conditions, PpIX localized in ER (Figure 1C), suggesting this organelle as its major target. In addition, ER-stress was associated with photodynamic effect (Figure 1D). These data postulated Me-ALA based-PDT as a potential Type-II ICD inducer.

ICD is a death mechanism in which specific stimuli lethally damage cancer cells while producing the spatiotemporally emission of immunogenic signals (15). Previous reports demonstrated the photodynamic mobilization of some of the main DAMPs involved in ICD, such as ATP (26), HMGB1 (24), and CRT (24, 25). In this sense, in the current work, we observed a significant translocation of CRT from ER to plasma membrane (Figure 2E). In an ICD context, this translocation of CRT occurs in a pre-apoptotic stage (before translocation of phosphatidylserine to the outer side of the plasma membrane) (39, 40). The ecto-CRT serves as a potent “eat me” signal for local patrolling DCs (39). For immunogenicity to be detected, dying cells must emit signals in addition to CRT. In fact, recently the capacity of surface-exposed CRT to initiate type I IFN-dependent anticancer immunity was shown (41). The immune response against cancer generated by type-I-interferons (IFN-1) has recently described. Exogenous and endogenous IFN-α/β have an important role in immune surveillance and control of tumor development. Accordingly, the role of TLR agonists as cancer therapeutic agents is being revisited with the idea of stimulating the production of endogenous type I IFN inside the tumor (31, 34, 36).

In addition, type-I-interferons (IFN-1) have recently emerged as novel DAMPs for the sequential events bridging innate and cognate immunity (16, 48). Both IFN-1 as well as ISGs had been activated *in vitro* and *in vivo* following anthracycline-based chemotherapy. It was described how the cancer autocrine axis of TLR3 > IFNs-I > IFNAR affects immunogenicity of anthracycline-mediated tumor cell death (14, 16). Based on these findings, IFN-1s are now classified as a Class IIIA DAMPs: endogenous native molecules operating as inducible DAMPs (49). Interestingly, to the best of our knowledge, the present study is the first to report the *in vitro* upregulation of IFN-1 expression in response to photodynamic treatment in melanoma (Figure 2). Our data suggest that Me-ALA based PDT stimulate the production of IFN-α/β and related ISGs through an autocrine molecular pathway (Figure 3).

For a successful immunogenic cell death promotion, the concomitant DAMPs must have activating effects on dendritic cells (DCs). DCs are mobile cells, and this feature is crucial for their antitumor action *in vivo* for the proper detection and capture of tumor antigens in peripheral tissues. Next, DCs upregulate the expression of co-stimulatory molecules, in order to cross-present and activate antigen-specific T cells. DAMPs recognition is an essential requirement for the activation of immature DCs associated with the expression of co-stimulatory T cell molecules (50). Here, we demonstrated that photosensitized melanoma promotion of both DCs migration to tumor site and DCs maturation was dependent on IFN-1 signaling (Figure 4). Taken together, our results show that cancer cells subjected to oxidative stress due to ER-associated pro-apoptotic PDT could potentiate antitumor immunity through an autocrine/paracrine activation of IFN-1 pathway.

In recent years, anticancer vaccination success has been correlated with the immunogenic potential of dead/dying cells used as antigen/adjuvant source. The danger signals-dependent efficacy of ICD-based DC vaccines has recently been shown (17, 51–54). However, chemical ICD inducers are not desirable for production of DC-based vaccines because they either leave residual drug concentrations behind or may exert cytotoxicity against DCs. For that reason, appropriate preselection of ICD should be critically considered (55–57). In this sense, as the prodrug Me-ALA is not toxic *per se* (Figure 1A), and given the immune stimulation properties observed (Figure 4), the PDT conditions here tested could represent a promising approach in the design of ICD-based DCs vaccines.

Collectively, our findings showed the effects of a novel danger signal released by PDT-treated cancer cells on the activation of DCs, emphasizing the potential relevance of PDT in adoptive/personalized immunotherapy protocols.

## Data Availability Statement

The datasets generated for this study are available on request to the corresponding author.

## Ethics Statement

The animal study was reviewed and approved by Comité de Ética de la Investigación de la Universidad Nacional de Río Cuarto (CoEdI).

## Author Contributions

ML: formal analysis, investigation, methodology, writing—original draft, funding acquisition, and project administration. FM, ER, and PA: formal analysis, investigation, and methodology. VR: conceptualization, supervision, resources, funding acquisition, and project administration. MM: conceptualization, formal analysis, resources, supervision, and writing—review and editing. NR: conceptualization, formal analysis, resources, supervision, funding acquisition, project administration, and writing—review and editing.

### Conflict of Interest

The authors declare that the research was conducted in the absence of any commercial or financial relationships that could be construed as a potential conflict of interest.
